# Clinical recovery of two hip adductor longus ruptures: a case-report of a soccer player

**DOI:** 10.1186/1756-0500-6-205

**Published:** 2013-05-22

**Authors:** Kristian Thorborg, Jesper Petersen, Michael Bachmann Nielsen, Per Hölmich

**Affiliations:** 1 Arthroscopic Centre Amager, Amager Hospital, Copenhagen University Hospital, Italiensvej 1, 2300 Copenhagen S, Hvidovre, Denmark; 2 Departments of Orthopaedic Surgery and Physical Therapy, Copenhagen University Hospital, Hvidovre, Denmark; 3 Arthroscopic Centre Amager, Copenhagen University Hospital, Hvidovre, Denmark; 4 Section of Radiology, Rigshospitalet, Copenhagen University Hospital, Copenhagen, Denmark; 5 Arthroscopic Centre Amager, Copenhagen University Hospital, Hvidovre, Denmark

**Keywords:** Hip adductor longus *ruptures*, Return to sport, Adductor strength, Ultrasonographic findings, HAGOS

## Abstract

**Background:**

Non-operative treatment of acute hip adductor longus ruptures in athletes has been described in the literature. However, very limited information concerning the recovery of this type of injury exists. This case represented a unique possibility to study the recovery of two acute adductor longus ruptures, using novel, reliable and validated assessment methods.

**Case presentation:**

A 22-year old male soccer player (Caucasian) sustained two subsequent acute adductor longus ruptures, one in each leg. The injuries occurred 10 months apart, and were treated non-surgically in both situations. He was evaluated using hip-strength assessments, self-report and ultrasonography until complete muscle-strength recovery of the hip adductors had occurred. The player was able to participate in a full soccer training session without experiencing pain 15 weeks after the first rupture, and 12 weeks after the second rupture. Full hip adductor muscle-strength recovery was obtained 52 weeks after the first rupture and 10 weeks after the second rupture. The adductor longus injuries, as verified by initial ultrasonography (10 days post-injury), showed evidence of a complete tendon rupture in both cases, with an almost identical imaging appearance. It was only at 6 and 10 weeks ultrasonographic follow-up that the first rupture was found to include a larger anatomical area than the second rupture.

**Conclusion:**

From this case we can conclude that two apparently similar hip adductor longus ruptures, verified by initial ultrasonography (10 days post-injury), can have very different hip adductor strength recovery times. Assessment of adductor strength recovery may therefore in the future be a useful and important additional measure for determining when soccer players with hip adductor longus ruptures can return safely to play.

## Background

Hip adductor longus ruptures are most commonly seen in football and ice-hockey [1,2]. Treatment of acute hip adductor longus ruptures in elite athletes includes both surgical and non-surgical approaches [3]. A case-series from 2009, including 19 American Football players, found that non-surgical treatment of proximal adductor tendon rupture results in faster return-to-play than surgical treatment in players competing in the National Football League [3]. Another advantage of non-operative treatment is that it avoids the risks associated with surgery, while still providing an equal likelihood of return-to-play [3]. However, that study did not include any assessment concerning the muscle-tendon-bone healing process or the muscle strength recovery of the adductors [3].

We have only identified one previous case-report, which has documented hip muscle strength and functional recovery after acute repair of a proximal adductor longus rupture by surgical re-fixation in two elite soccer players [1]. This study showed normalised hip adductor strength after 8 weeks and return to previous competitive level 10 weeks post-surgery. However, muscle strength was assessed by manual muscle testing which should be considered cautiously, as manual muscle testing has been shown to fail the identification of substantial muscle strength deficits [4-6]. Therefore, crucial aspects of the adductor muscle recovery process after an adductor longus rupture are largely unknown [3].

In the present case, we report on a 22-year-old male, playing soccer at a sub-elite level, who sustained two acute hip adductor longus ruptures, one in each leg, within a 10-month interval. This case represented a unique possibility to study the recovery of these ruptures, using novel, reliable and validated assessment methods [7].

## Case presentation

### Case description - mechanism of injury (Adductor rupture 1 and 2)

**Figure 1:**
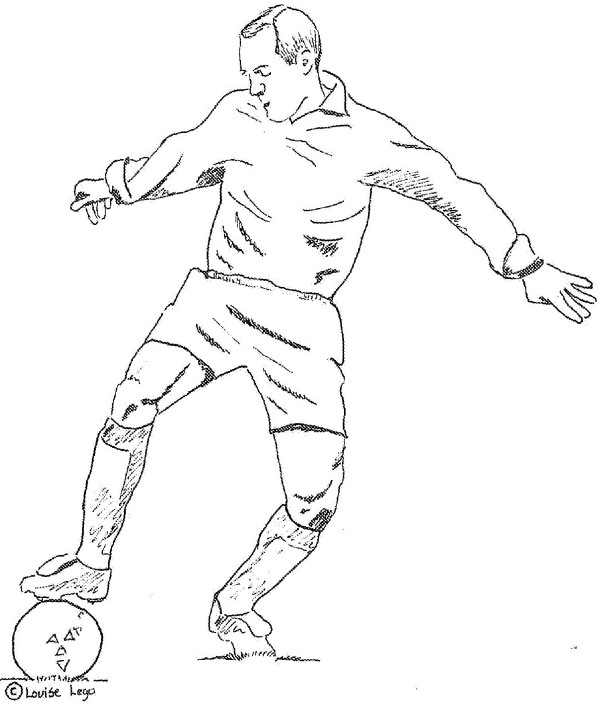
Mechanism of injury for adductor longus rupture.

The player described the first injury (left leg) as occurring in a one-on-one attacking situation. When trying to get pass a defender, dribbling with the ball, he was pushed from the side by the defender. While being pushed, the player tried to maintain balance and ball possession by reaching for the ball with his right leg, while at the same time having all his weight shifted onto the left leg due to the push. He described the injured left hip as being in a position of abduction, extension and externally rotation, while the upper body was forced laterally towards the side of the left hip (Figure 1). He felt a sudden pop and a sharp pain, fell to the ground, and was unable to continue playing. The second injury (right leg) occurred in an almost identical situation 10 months later. The player had no history of previous injuries in the hip and groin.

### Clinical examination

**Figure 2:**
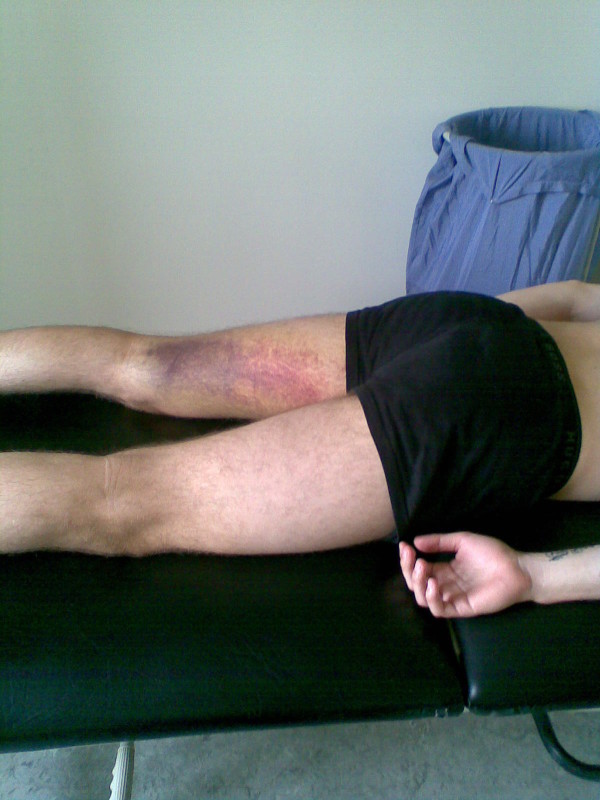
**Haematoma on the posterior medial thigh 2 weeks after adductor longus rupture.** Adductor longus rupture 1 (left leg).

**Figure 3:**
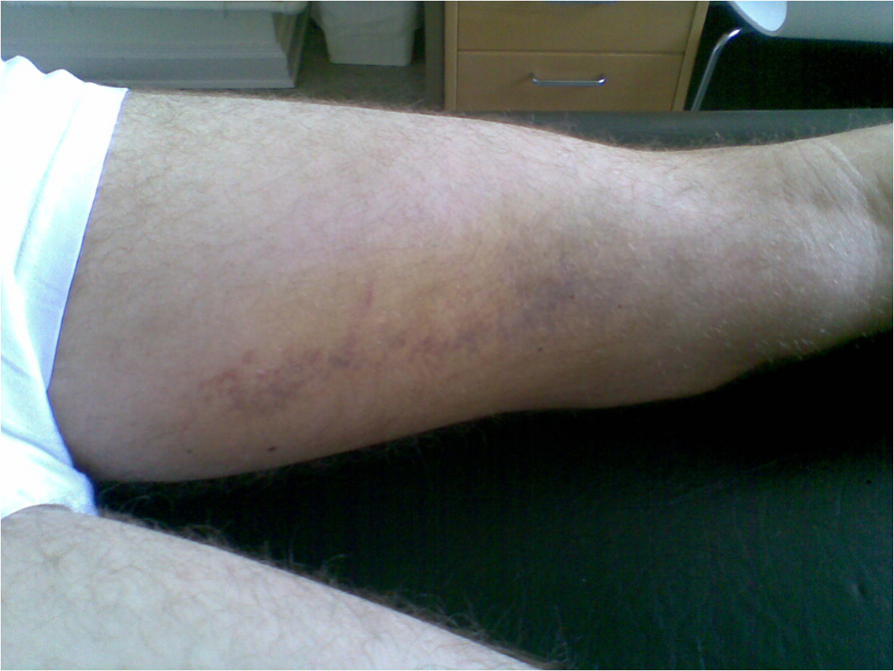
**Haematoma on the posterior medial thigh 2 weeks after adductor longus rupture.** Adductor longus rupture 2 (right leg).

Ten days after both injuries, the player presented for clinical consultation. The clinical examination was performed by a physiotherapist (KT). The examination revealed significant swelling in the proximal groin region near the pubic bone, and a large haematoma was spreading down the medial and posterior thigh for both injuries. The first rupture (Figure 2), however, was much more visible at the medial and posterior aspect of the thigh than the second rupture (Figure 3), indicating that more bleeding had occurred after the first rupture.

Acute and severe pain was present on palpation and isometric contraction of the adductor longus muscle-tendinous complex at the proximal insertion site. The hip joint could not be examined during the initial visits due to pain restricting the assessment of full range of motion. The hip was clinically examined 6 weeks post injury in both cases. The clinical examination of the hip joint at 6 weeks did not show any severe restriction of passive hip movement or sign of any additional intra-articular injury, and indicated that both injuries were adductor-related [8,9]. However the specific location and extent of the injury could not be determined. The player was therefore referred to diagnostic imaging. An ultrasonographic examination was performed by a radiologist (MBN) with extensive experience in ultrasound of the hip and groin [10]. The scan revealed an isolated complete adductor longus rupture in both cases, with no additional injury to the iliopsoas, rectus abdominis, conjoined tendon, inguinal canal or other relevant structures.

## Clinical assessment of the recovery

### Hip strength assessment

Isometric hip muscle strength assessment was performed by the examining physiotherapist (KT) using standardised and reliable strength measurements [11]. Hip muscle strength testing was continued until no difference (≤ 5%) in hip adduction strength was identified between the affected leg and the reference leg [12]. For the first injury (left leg), baseline strength measures of the contralateral side (right leg) were determined two weeks post trauma, and these data were used as a reference for normalisation of hip muscle strength [12]. For the second injury (the right leg), the aforementioned baseline strength measures for the ipsilateral side (right leg), obtained 10 months earlier, were used as the reference for hip muscle strength normalisation. Strength testing was used to monitor the loss and recovery of hip muscle strength.

### Ultrasonographic examination

Ultrasonography was repeated to describe the anatomical healing over time. The following parameters were described: presence of blood/fluid; muscle/tendon injury; calcifications (myositis ossificans) and hyperemia/inflammation based on increased color Doppler activity.

### Self-reported activity level

Self-reported pain-free activity level was reported at a weekly basis. The player was asked when he was able to perform the following activity pain-free, in this sequence: 1) walk, 2) jog, 3) run, 4) sprint, 5) make sudden changes in direction, 6) kick and 7) participate in a full training session (Table 1). He was only allowed to proceed to the next activity level when the preceding activity was reported as being pain-free, to ensure a safe progression and return-to-play.

**Table 1:** Pain-free activity level reported at a weekly basis

	**Adductor rupture 1**	**Adductor rupture 2**
Walking	2 weeks	2 weeks
Jogging	4 weeks	5 weeks
Running	5 weeks	6 weeks
Sprinting	11 weeks	8 weeks
Sudden change of direction	12 weeks	10 weeks
Kicking	13 weeks	11 weeks
Full training session	15 weeks	12 weeks

### New and recurrent injury registration

The player was contacted by phone or e-mail on a weekly basis during the testing period. Injuries and recurrent injuries were documented, according to the FIFA consensus statement on injury definitions [13,14]. A retrospective three-year follow-up period reporting injuries and recurrent injuries was performed.

### Long-term follow up

At two- (Adductor rupture 2) and three-year (Adductor rupture 1) follow-up, self-reported disability; Copenhagen Hip And Groin Outcome Score (HAGOS) [15], muscle strength [11,12], and ultrasonography were performed [10].

### Management

Both injuries were treated non-surgically, since this seems to provide early return to play with no additional risk of recurrence [3]. At the initial consultations a supervised exercise program was introduced to the player. The exercise program was based upon the exercise protocol described by Holmich et al. [16], developed for patients with longstanding adductor-related groin pain. This program was followed 3–5 times weekly. Additional strengthening exercises with an eccentric emphasis, using strength training machines, were added for hip-adductors, -abductors, -flexors and abdominal muscles. Strength training in machines was initiated after 10 weeks, starting with a relative load of 20 repetitions maximum (RM) progressing into 15, 12 and 10 RM, when these loads could be tolerated and executed pain-free. Soccer-specific exercises, including running and kicking exercises, were added subsequently when these activities were pain-free, following the predefined algorithm for activity progression (Table 1).

Return to a soccer match was not advised until all soccer-specific activities could be performed pain-free, with no aggravation of symptoms the following day(s), and when the hip adduction strength difference between the injured leg and the reference leg was <10% (± 5%) [2,11,12].

## Results

### Self-reported activity level

The player was able to participate in a full pain-free soccer training session 15 weeks after the first adductor rupture, and 12 weeks after the second adductor rupture (Table 1).

### Hip strength assessment

**Figure 4:**
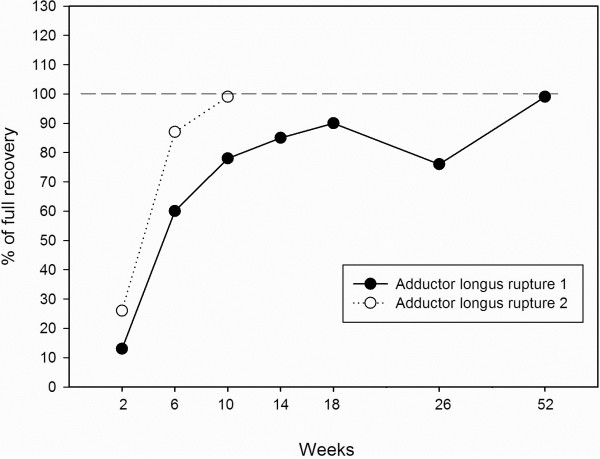
Hip adduction strength recovery after adductor longus ruptures.

**Figure 5:**
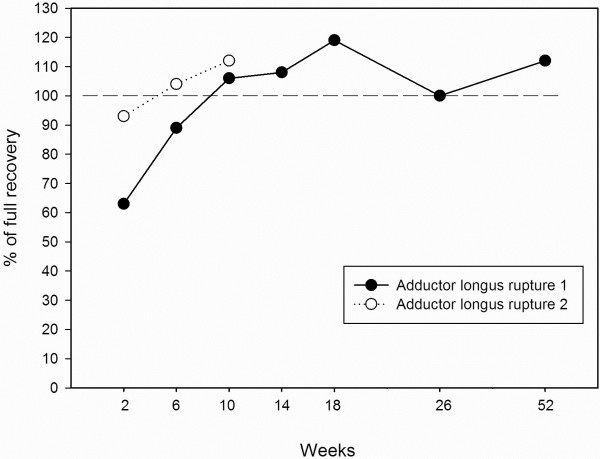
Hip flexion strength recovery after adductor longus ruptures.

Figures 4 and 5 shows the strength recovery of hip adductors and hip flexors during the initial 52 weeks after the injuries. For the first rupture hip adduction strength was only 90% recovered 18 weeks after the injury, and normalised somewhere between 6 months and 1 year after the injury. For the second rupture hip adduction strength was normalised after 10 weeks.

### Ultrasonographic examination

#### Adductor rupture 1

**Figure 6:**
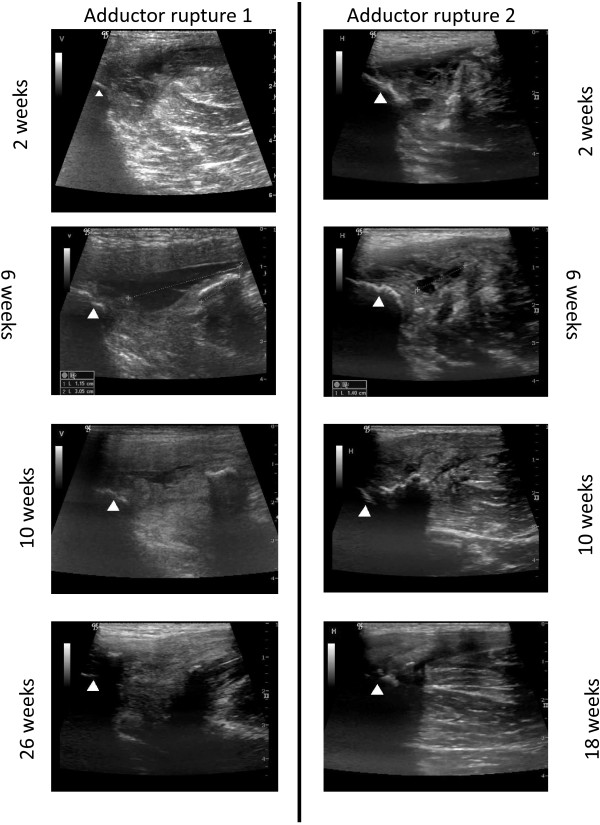
**Ultrasonographic assessment and time of anatomical recovery following adductor longus ruptures 1 (AR 1) and 2 (AR 2).** AR 1 (2 weeks): White arrow (Insertion), Snip of torn proximal adductor longus tendon, Haematoma, Distracted muscle. AR 2 (2 weeks): White arrow (Insertion), Distracted muscle, Fluid / Haematoma. AR 1 (6 weeks): Distracted muscle, Calcification, Fluid. AR 2 (6 weeks): Calcifications, Fluid. AR 1 (10 weeks): Distracted muscle, Calcification, Fluid. AR 2 (10 weeks): Calcifications. AR 1 (18 weeks): Calcification. AR 2 (26 weeks): Calcification.

At 2 weeks the ultrasonographic examination showed a full adductor longus rupture at the proximal musculoskeletal junction with hematoma in the proximal 3–4 cm. After 4 weeks several calcifications in the size of approximately 4 mm were present in close relation to the insertion of the adductor longus tendon as well as within the tendon. Significant color Doppler activity representing hyperemia (inflammation) was present in a 6 cm long focal area from the insertion of the tendon. After 6 weeks the calcifications had increased in size to 5 to 22 mm per element and were still found near the insertion. Furthermore, a 3 cm long and 1 cm wide fluid-filled area had developed and color Doppler activity was still present. Fluid and color Doppler activity gradually decreased during the following 12 weeks. Ultrasonographic findings after half a year and beyond were unchanged. Calcifications were seen at the insertion and within the tendon, and the tendon was thickened from the site at insertion and 6 to 7 cm distally (Figure 6).

#### Adductor rupture 2

At 2 weeks the ultrasonographic examination showed a full adductor longus rupture at the proximal musculoskeletal junction and focal fluid-filled areas near the insertion. After 6 weeks several calcifications were present in immediate relation to the insertion of the adductor longus tendon. The proximal 4 to 5 cm of the tendon was thickened and significant color Doppler activity representing hyperemia (inflammation) was present in this area. A 1.5 cm long and 0.5 cm wide fluid-filled area was seen. Fluid and color Doppler activity gradually decreased during the following 12 weeks, but calcifications persisted in the proximal part of the tendon and were still seen 2 years after the injury (Figure 6).

### New and recurrent injury registration

No new or recurrent injuries occurred in the hip or groin region after the initial adductor ruptures in each leg.

### Long-term follow up

**Figure 7:**
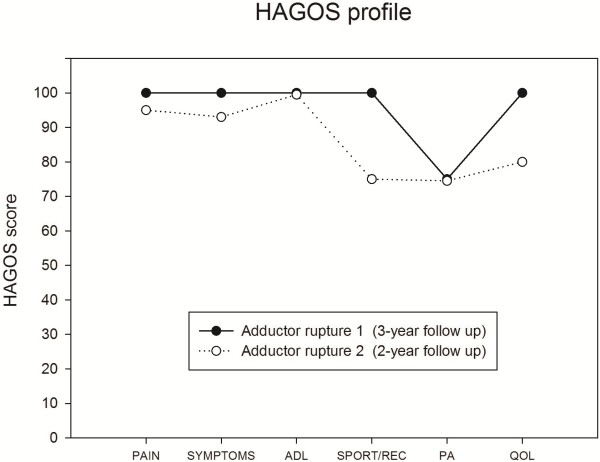
Hip and Groin Outcome Score (HAGOS) at 2-year and 3-year follow-up after adductor longus ruptures.

**Figure 8:**
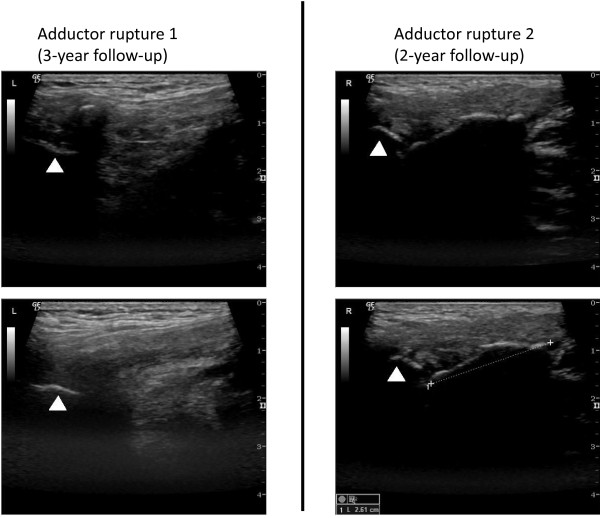
**Ultrasonographic assessment at 2-year and 3-year follow up following adductor longus ruptures.** Adductor rupture 1 (3 years): White arrow (Insertion), Small calcification in the tendon. Adductor rupture 2 (2 years): White arrow (Insertion), Calcifications (2.6 cm).

At two-year (adductor rupture 2) and three-year follow-up (adductor rupture 1) the self-reported disability score (HAGOS) [15] showed that the ability of the player to participate in his preferred physical activity (soccer) was a little affected for both injuries (Figure 7). Hip muscle strength was still normalised at long-term follow-up. At two-year follow-up, the ultrasonographic examination revealed calcification of the tendon (second rupture) near its insertion site, extending 2.6 cm along the muscle-tendinous junction (Figure 8).

## Discussion

The present case-report shows that two apparently very similar adductor longus ruptures can have very different hip-adductor strength recovery times. In the present study hip adduction strength was restored after 1 year for the first rupture, and after 10 weeks for the second rupture.

### Ultrasonographic findings and adductor muscle-strength recovery times

The most interesting imaging finding was that both adductor longus injuries, as verified by initial ultrasonography (10 days post-injury), had an identical imaging appearance, showing a similar complete tendon rupture. Only at 6 and 10 weeks follow-up, ultrasonography indicated that the first rupture included a larger anatomical area compared to the second rupture.

### Preventing re-injury

Previous groin-injury exposes soccer players to a 5-fold increased risk of a future groin injury [14]. It has been suggested that inadequate rehabilitation may explain this phenomenon [17]. It therefore seems of great importance to optimize the rehabilitation program, improving hip adductor function and its eccentric strength capacity, since muscle injuries and recurrent muscle injuries have been related to eccentric hip muscle weakness [18-21]. In this case no recurrent adductor injuries were encountered for any of the initial adductor ruptures. There may be several explanations for the positive outcome of the rehabilitation in terms of no recurrent injuries. Firstly, the thorough monitoring of hip-adductor strength made it possible to measure when hip adductor strength was normalized, before advising return-to-play. A rehabilitation period of 12–15 weeks before return-to-play is a fairly long rehabilitation period, compared with previous studies concerning hip adductor longus ruptures [1,3]. It could be argued that a recovery period of 15 weeks would be difficult to accept by the player, the coach or the club. However, the advantage of the hand-held dynamometer measurements is that they provide precise insight into the muscle strength recovery. This gives the sports practitioner an objective impression of adductor muscle function and recovery instead of solely having to rely on subjective reports, from the player, regarding pain during sports-specific activity as a guideline for return-to-play [11,12]. In fact, for both injuries the player reported no pain during running after 35–38 days (5–6 weeks). For players and coaches this would often indicate that the player is nearly ready for “return-to-play”. However, since muscle strength for the first rupture at the stage where he was running pain-free (6 weeks) was only 60% of the reference leg, we advised the player not to return to soccer-play at this point.

### Long-term follow of a hip-adductor longus rupture

At the long term follow-up the player reported that the second injury from time to time would cause him minor problems during soccer play, and the HAGOS scores seemed to support this notion. Based on knowledge of injuries to the hamstring muscle-tendinous complex, as estimated by MRI, involvement of the proximal free hamstring tendon and proximity to the ischial tuberosity are associated with longer time to return to pre-injury level than injuries with no proximal tendinous involvement [22]. The long term ultrasonographic follow-up assessment in the present study seems to indicate that while being less severe in terms of bleeding and swelling, the second rupture may have occurred near the insertion point (enthesis), possibly including a few intact - but pain eliciting - insertional fibers, and/or pain eliciting scar tissue formation at the muscle-tendinous insertion point (enthesis) [23]. Both scenarios that could possibly explain the minor difference in long term self-reported disability between the two injuries [22,23].

## Conclusion

From this case we can conclude that two apparently similar hip-adductor longus ruptures, verified by initial ultrasonography (10 days post-injury), can have very different hip-adductor strength recovery times. Assessment of hip-adductor strength recovery may therefore in the future be a useful and important additional measure for determining when soccer players with hip adductor longus ruptures can return safely to play.

## Consent

Written informed consent was obtained from the patient for publication of this Case report and any accompanying images. A copy of the written consent is available for review by the Editor of this journal.

## Competing interests

The authors declare that they have no competing interests.

## Authors’ contribution

KT drafted the manuscript. KT and PH were responsible for the study concept and design. Acquisition of data was performed by KT, JP and MBN. All authors were responsible for the interpretation of data and critically revised the manuscript. All authors read and approved the final manuscript.

## Authors’ information

Jesper Petersen, Michael Bachmann Nielsen and Per Hölmich are co authors.
